# Cytotoxic effects of four different root canal sealers on human osteoblasts

**DOI:** 10.1371/journal.pone.0194467

**Published:** 2018-03-26

**Authors:** Susanne Jung, Sonja Sielker, Marcel R. Hanisch, Viktor Libricht, Edgar Schäfer, Till Dammaschke

**Affiliations:** 1 Department of Cranio-Maxillofacial Surgery, Research Unit Vascular Biology of Oral Structures (VABOS), University Hospital Münster, Münster, Germany; 2 Department of Periodontology and Operative Dentistry, Westphalian Wilhelms-University, Münster, Germany; 3 Central Interdisciplinary Ambulance in the School of Dentistry, Münster, Germany; National Taiwan University, school of dentistry, TAIWAN

## Abstract

The aim of this study was to evaluate the effect of an epoxy resin-based (AH-Plus), a zinc oxide eugenol containing (Pulp-Canal-Sealer) and two calcium silicate containing (MTA-Fillapex and BioRoot-RCS) sealers on primary human osteoblasts (hOB) in freshly mixed and set state. All sealers were mixed strictly according to the manufacturers´ instructions and identically samples were produced. In a pretest cytotoxic sealer concentrations were determined. Thus, for the main cell culture study, dilutions of sealer extract 1:1, 1:2, and 1:10 were used. To simulate a clinical scenario, extracts from freshly mixed sealer were added to the cells on day one. Extracts form set sealers were used for subsequent culturing for 24h, 7d, 14d, and 21d. Cell viability was analyzed by living-cell-count, MTT-assay, and living/dead-staining, cytotoxicity by LDH-assay, and changes by Richardson-staining. All data were statistically evaluated by one way ANOVA and a posthoc analysis with Bonferroni-Holm testing (*p*<0.05). AH-Plus was cytotoxic in a freshly mixed state, but not when the sealer was set. MTA-Fillapex and Pulp-Canal-Sealer were cytotoxic in a fresh as well as in a set state. BioRoot-RCS showed the lowest toxicity in both states; where as a regeneration of the cells could be observed over time (*p*<0.05). Contact of freshly mixed AH-Plus to osteoblasts should be avoided. Pulp Canal Sealer and MTA-Fillapex showed no biocompatibility in contact with osteoblasts at all. BioRoot-RCS had a positive influence on the cell metabolism (bioactivity) and is biocompatible.

## Introduction

The aim of a root canal filling is the three dimensional bacteria and fluid tight seal of the entire root canal system in order to prevent passage of microorganisms from coronal to apical or vice versa [1, 2]. Furthermore, root canal filling materials should be more or less insoluble to prevent dissolving by body fluids in the root canal. It is well known, that breakdown products from root canal sealers may have an adverse effect on the proliferative capability of periradicular cell populations [3]. It has to be kept in mind that, besides the apical foramen, numerous microscopic and macroscopic communications exist between the root canal system and the periodontal ligament and the surrounding bone, namely dentinal tubules, accessory foramina and lateral canals [4]. Thus, tissue fluid can easily penetrate the root canal system, resulting in degradation of the sealer material and subsequent leaching out various components. Leached substances may then migrate to the periodontal tissues and alveolar bone, generate local periapical inflammatory reactions and adverse effects [5, 6]. If sealer and sealer components come into direct contact with periradicular tissues over extended periods of times, they may cause irritation and may result in delayed wound healing [7]. In addition, overfilled sealer can directly interact with adjacent tissues [5]. Sealer extruded into the periradicular tissue can be highly irritating [1].

Freshly mixed sealers applied in the root canal become immediately involved in local eluation processes because of the contact with extracellular fluids. The contact of the eluents with the periradicular tissue is concentration- and time-dependent and effect bone metabolisms and regeneration [8]. Hence, since many decades it has been claimed that sealer should be biocompatible and well tolerated by the periradicular tissue [9]. Nevertheless, until today, it is stated in literature that all root canal sealers (regardless of the type) exhibit toxicity in their freshly mixed state, but on setting, their toxicity is greatly reduced and most sealers become relatively inert [1, 2].

To overcome the problem of cytotoxicity, recently tri- or respectively di- and tricalcium silicate based root canal sealers were developed as an offspring of di- and tricalcium silicate cements (e.g. mineral trioxide aggregate; MTA). These di- and tricalcium silicate cements were introduced in dentistry e.g. for the repair of lateral root perforations and retrograde root end fillings [10]. It is well known, that di- and tricalcium silicate cements are highly biocompatible and bioactive. However, there are only few data about cytotoxicity or biocompatibility of the new calcium silicate-based sealers [11–14]. Sealer should be biocompatible and well tolerated by the periradicular tissue, but it remains unclear whether these new sealers are really an improvement in terms of biocompatibility compared to conventional sealers. This is an issue worthy to be discussed in dental research and clinical aspect. Thus, the aim of the present study was to evaluate the cytotoxicity of four different sealers (two new calcium silicate based and two conventional) to human osteoblasts in an unset and set condition. The hypothesis tested was that all sealers perform equally with regard to the effect on human osteoblasts.

## Material and methods

### Primary human osteoblast

Human cancellous bone was obtained from the Department of Craniofacial Surgery, University Hospital, Münster (Germany). Primary osteoblasts were harvested from tissue that was collected anonymously from leftover tissue from bone chips collected during modelling mandibular osteotomies or the surgical removal of lower wisdom teeth.

All participants provided their written informed consent to participate in this study. The Ethical Committee of the Westphalian Wilhelms-University approved the use of human cells (Reg. No. 2010-462-t). The handling of all human samples followed strictly the “Declaration of Helsinki”. The human cells were harvested and cultured according to a standardized protocol.

Bone pieces were washed up to 5 times with Dulbecco´s Phosphate Buffert Saline (DPBS; D8537, Sigma-Aldrich, Munich, Germany) to remove blood and debris. Bone pieces were crushed into small bone particles with a Luer Hohlmeissel forceps (Aesculap, Tuttlingen, Germany) and placed in cell culture dish. Then, the bone particles were cultured in MM0 medium (HGEM; High Growth Enhancement Medium, 0912337, MP Biomedicals, Eschwege Germany) containing 12% fetal bovine serum (FBS, S0615) and 1% of penicillin [10.000 U/ml] / streptomycin [10.000 μg/m], 1% amphotericin b [250 μg/ml], and 1% L-glutamine [200 mM] (A2212, A2612, K0282, Biochrom, Berlin, Germany).

Outgrowth of cells was checked for the first time after 7 days to minimize particle floating. After that outgrowth was controlled daily and bone particles were removed after 14 days. From this time point 0.004% Fortecortin (2 mg/ml; Merck Pharma, Darmstadt, Germany) for osteogenic differentiation was added to MM0. Cells were cultivated at 37 °C with 5% CO_2_, while being fed every 5 to 7 days and passaged after reaching nearly total confluence. The outgrowing cells were characterized immunohistochemically by positive expression of osteocalcin, osteonectin and collagen I. Mineralization of outgrowth cells were induced by adding 10 mM ß-glycerophosphate and 0.14 mM L-ascorbic acid (G9891, A4544, Sigma-Aldrich, Munich, Germany) to MM0 medium. Cells were cultivated for nearly 28 days. Calcium was stained with an alizarin red S solution (40 mM, Sigma-Aldrich, Munich, Germany).

The second passage was used for the experiments. Osteoblasts were seeded with a concentration of 5.300 cells / cm^2^ in 24-well culturing plates (TPP, Trasadingen, Switzerland) and were allowed to adhere for 24 h. The cells were cultured in their respective cell culture medium HGEM.

### Sealers

Following sealers were used in the present study: AH Plus (Dentsply DeTrey, Konstanz, Germany), Pulp Canal Sealer (Kerr, Scafati, Italy), MTA Fillapex (Angelus, Londrina, Brazil), and BioRoot RCS (Septodont, St. Maur-des-Fossés, France). These commercial products are all use in dental area. AH Plus is a conventional epoxy resin-based root canal sealer and is widely used and well investigated [15–17]. Therefore, it served as control group in this study. Pulp Canal Sealer is a zinc oxide-eugenol sealers and it is well known that these types of sealers possess marked cytotoxic and tissue-irritating potencies in *ex vivo* cell culture studies and are characterized by a high cytotoxic potency [5].

MTA Fillapex and BioRoot RCS are both comparably new root canal sealers and are both calcium silicate based. Nevertheless, the composition is quite different. Whereas, MTA Fillapex is a salicylate-resin material that contains 13.2% set MTA particles, BioRoot RCS is composed mainly from a tricalcium-silicate powder that has to be mixed with water [18, 19].

To produce identical sealer samples, all sealers were mixed according to manufactures´ information and applied into silicone molds (diameter 4 mm, height 1.5 mm, volume 18.85 mm^3^). From all sealers 20 specimens were produced. To ensure complete setting of all sealers, samples were immersed in physiological solution (Hank´s balanced salt solution) at 37 °C for 48 h [20]. The proper setting was evaluated in a pretest. After setting, the materials were weighed (accuracy ± 0.0001; Sartorius 1801MPS, Göttingen, Germany) three times and the average reading was recorded. The mean weights of test specimens with identically volume were for AH Plus 47.6 mg (± 1.3 mg), MTA-Fillapex 31.6 mg (± 1.3 mg), Pulp Canal Sealer 49.4 mg (± 1.9 mg), and for BioRoot RCS 37.3 mg (± 1.5 mg). The mean weight of one test body for each sealer was defined to be the one fold concentration (single-strength dilution) of the cell culture medium in mL, in which the appropriate sealer was stored.

### Determination of cytotoxic sealer concentrations

To evaluate suitable sealer eluate concentration, all sealers were mixed under sterile conditions and added to the medium (MM0 medium, High Growth Enhancement Medium; MP Biomedicals, Eschwege, Germany) without any supplements. To produce sealer eluates, the medium suspension was incubated at 37 °C for 24 h in contact with the sealer samples. After that, the supernatant liquid was filtrated under sterile conditions and stored at minus 20 °C until use. Extracts with 4-fold and also single-strength concentration were mixed and diluted to lower concentrations: 4:1, 2:1, 1:1, 1:2, 1:5, 1:10, 1:20, 1:100 dilutions of the cell culture medium were used to determine those concentrations in which the cells will survive. Two kinds of sealer extracts were produced: extracts from freshly mixed or from set sealer. This resulted in (4 different sealers x 8 dilutions x 2 [fresh & set sealer]) 64 cell cultures. These 64 cell cultures were then studied in triplicates (n = 192). Alteration of pH induced by added sealer in culturing medium was measured with a pH meter (inoLab pH 7110, WTW, Weilheim, Germany).

In contact to extracts from Pulp Canal Sealer cells survived in a dilution of 1:2 (24.7 mg/ml). No differences between extracts from freshly mixed and set sealer were observed. In contact to extracts from freshly mixed MTA Fillapex cells survived in a dilution of 1:2 (15.8 mg/ml) and one fold concentration from set MTA Fillapex (31.6 mg/ml). Cell survived in contact to extract from freshly mixed BioRoot RCS in a dilution 1:10 (3.7 mg/ml) as well as in a 10-fold higher concentration from set BioRoot RCS (37.3 mg/ml). Extract from freshly mixed AH Plus was cytotoxic. Cells survived only in the lowest tested dilution of 1:100 (0.48 mg/ml). In contrast, extract from set AH Plus had no cytotoxic effects. Cells survived even in a concentration of 4:1 (190.4 mg/ml).

For all sealer extracts no marked changes of the pH value of the culturing medium were observed, except for BioRoot RCS. Only in the BioRoot RCS samples with a concentration of 4:1 an increase of the pH value to 11 was detected during the first 24 h.

### Cell culture studies with sealer extracts

Due to the determined cytotoxic concentrations, in the following main cell culture study dilutions of sealer extract 1:1, 1:2, and 1:10 were used from freshly mixed or set sealer. Osteoblasts were seeded with a concentration of 5.300 cells/cm^2^ in 24-well culturing plates and were allowed to adhere for 24 h. To simulate a clinical scenario, extracts from freshly mixed sealer were added to the cells on day one. Extracts form set sealers were used for subsequent culturing and renewed every week. The pH value of the culturing medium was measured using a pH meter (inoLab pH 7110, WTW, Weilheim, Germany). The cell culture studies were done in triplicates.

After 24 h, 7 days, 14 days, and 21 days cell viability (living cell count; MTT; living dead staining), cytotoxicity (LDH assay) and changes in cell morphology (Richardson staining) were analyzed. This resulted in (4 different sealers x 3 dilutions x 2 [fresh & set sealer] x 4 time intervals) 96 experimental groups and 288 cell cultures. Cell growth without sealer extracts in culturing medium (n = 12 cultures [4 time intervals in triplicates]) was used as control.

### Cell viability

Living cell count was performed with the CASY1 cell counter (Schärfe System, Reutlingen, Germany). Cell proliferation rates were estimated with a MTT assay. The conversion of the yellow thiazolyl blue tetrazolium bromide (0.5 mg/ml; Sigma-Aldrich) to the purple formazan by the cellular NAD(P) reflux was measured at λ 570 nm. Cytotoxic effects were determined with the Pierce LDH Cytotoxicity Assay (ThermoFisher Scientific, Waltham, MA, USA). All assays were performed according to manufacturers’ protocols and done in triplicates.

The qualitative analysis of cell viability was performed via fluorescein diacetate / propidium iodide (FDA/PI) staining, where FDA (Sigma Aldrich) stains viable cells green, and PI (Fluka, Darmstadt, Germany) stains necrotic and apoptotic cell nuclei red.

### Richardson staining

For histological evaluation the cell cultures were fixed in methyl ethanol (Merck, Darmstadt, Germany), air died, and a Richardson staining was performed. The staining solution I contained 1% methylene blue (Merck) in 1% sodium borate (Merck). The staining solution II contained 1% azure in distilled water. Both solutions were mixed 1:1 bevor use.

### Statistical analysis

Statistical analysis was carried out by one way ANOVA using a modified Levene testing and *p* < 0.05, and a posthoc analysis with Bonferroni-Holm testing (Daniel’s XL Toolbox version 6.53; http://xltoolbox.sourceforge.net).

## Results

### Main cell culture tests

**Fig 1:**
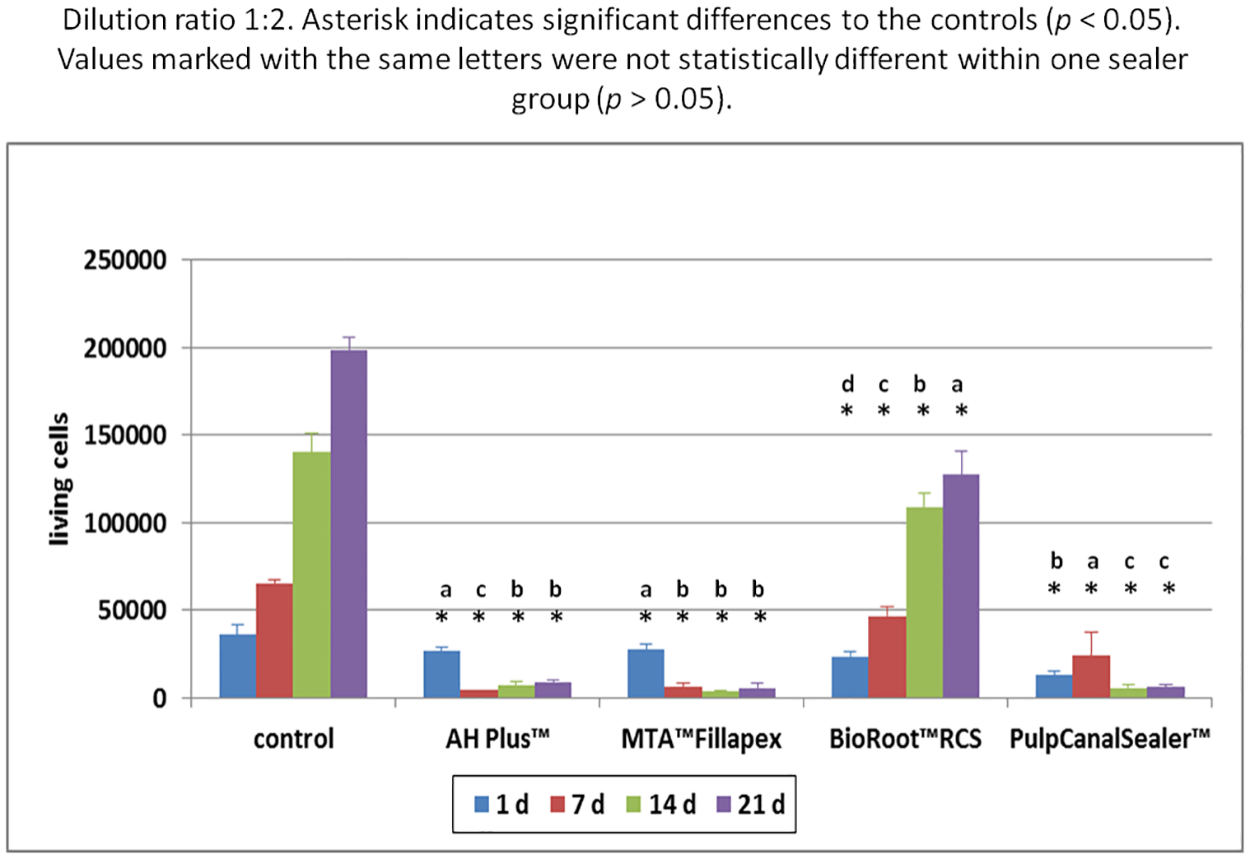
Quantity of living human osteoblasts after contact to different endodontic sealers (dilution 1:2) up to 21 d. Asterisk indicates significant differences to the controls (*p* < 0.05). Values marked with the same letters were not statistically different within one sealer group (*p* > 0.05).

**Fig 2:**
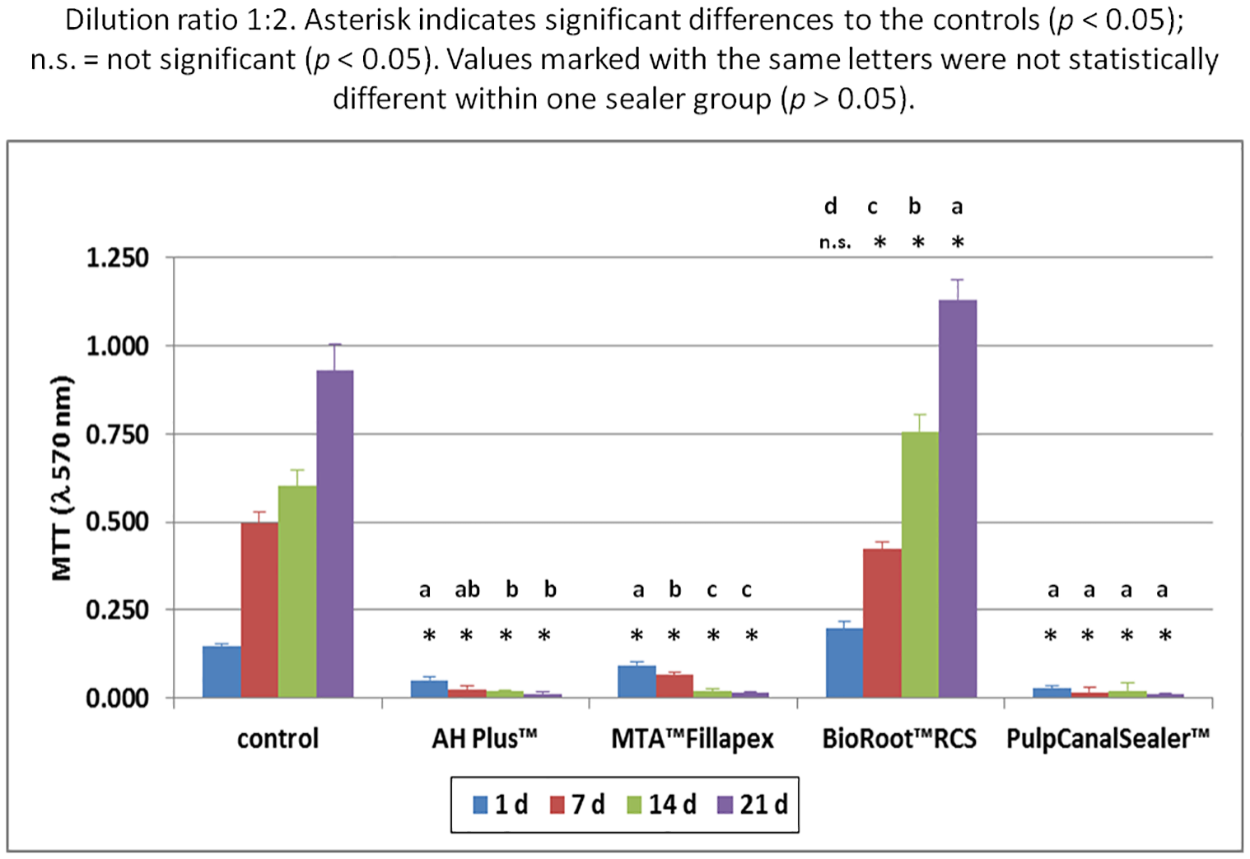
MTT assay of human osteoblasts after contact to different endodontic sealers (dilution 1:2) up to 21 d. Asterisk indicates significant differences to the controls; n.s. = not significant (*p* < 0.05). Values marked with the same letters were not statistically different within one sealer group (*p* > 0.05).

**Fig 3:**
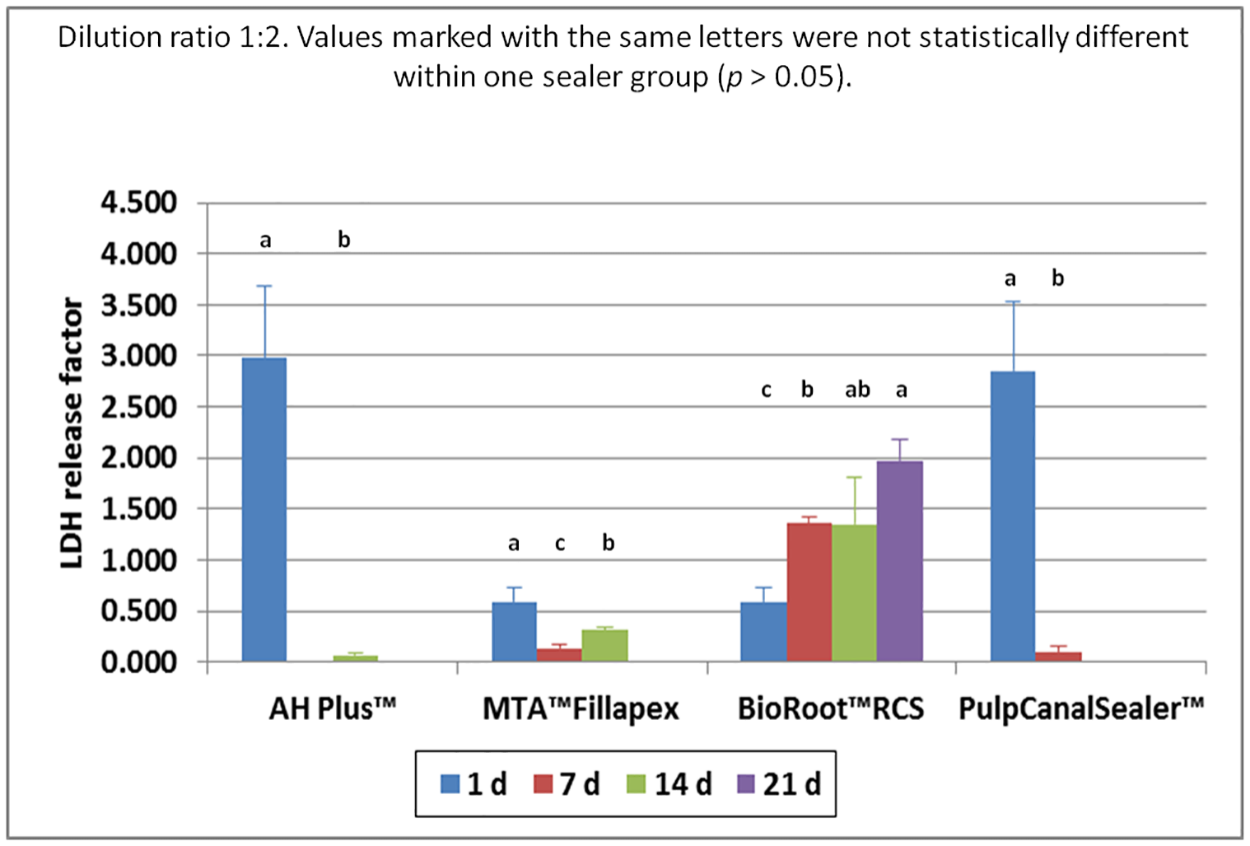
LDH release factor of human osteoblasts after contact to different endodontic sealers in correlation to control group (dilution 1:2) up to 21 d. Values marked with the same letters were not statistically different within one sealer group (*p* > 0.05).

**Fig 4:**
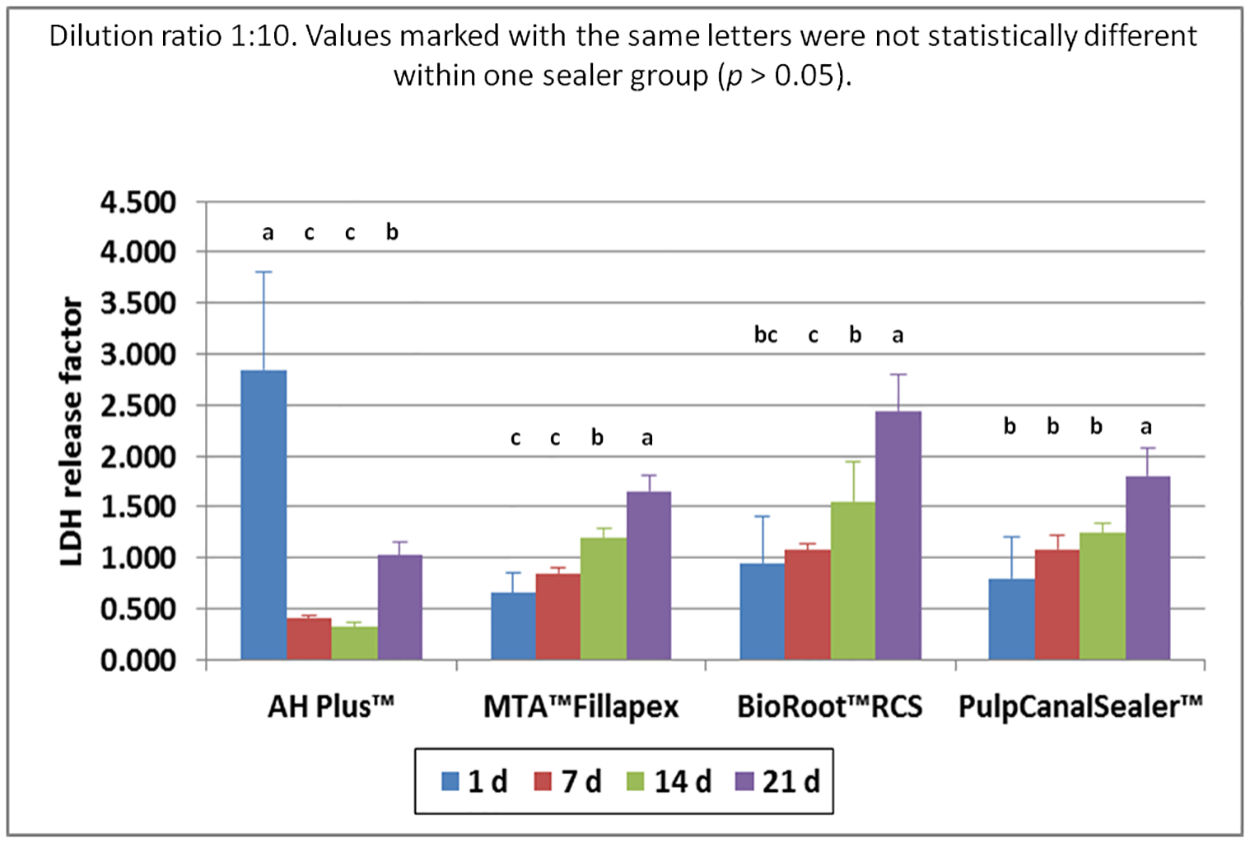
LDH release factor of human osteoblasts after contact to different endodontic sealers in correlation to control group (dilution 1:10) up to 21 d. Values marked with the same letters were not statistically different within one sealer group (*p* > 0.05).

Based on the preliminary study, sealer extracts in a dilution of 1:10, 1:2 and 1:1 were used for the main cell culture test (n = 288). Within the one fold concentration (n = 96 cell tests), all cells died during the first days irrespective of sealer type. At a sealer extract dilution of 1:10 (n = 96 cell tests) all cells in the test groups showed the same survival rate as the cells in the control group, with the exception of AH Plus. Here the cell survival rate was lower. The count of living cells for the dilution 1:2 (n = 96 cell tests) are summarized in Fig 1 and the results of the MTT assay in Fig 2. The results of the LDH assay for the 1:2 are summarized in Fig 3 and for the 1:10 dilution in Fig 4.

One way ANOVA was performed for each assay and differences were analyzed on a level of significance of *p* < 0.05, with regard to living cell count (*p* = 0.0007) proliferation rate (*p* = 0.0024), and cytotoxicity (*p* = 0.002). Significant differences were obtained. Statistically significant differences (*p* < 0.05) within the individual seal groups and in comparison to the controls at the different examination times are indicated in the figures. (Figs 1–4)

**Fig 5:**
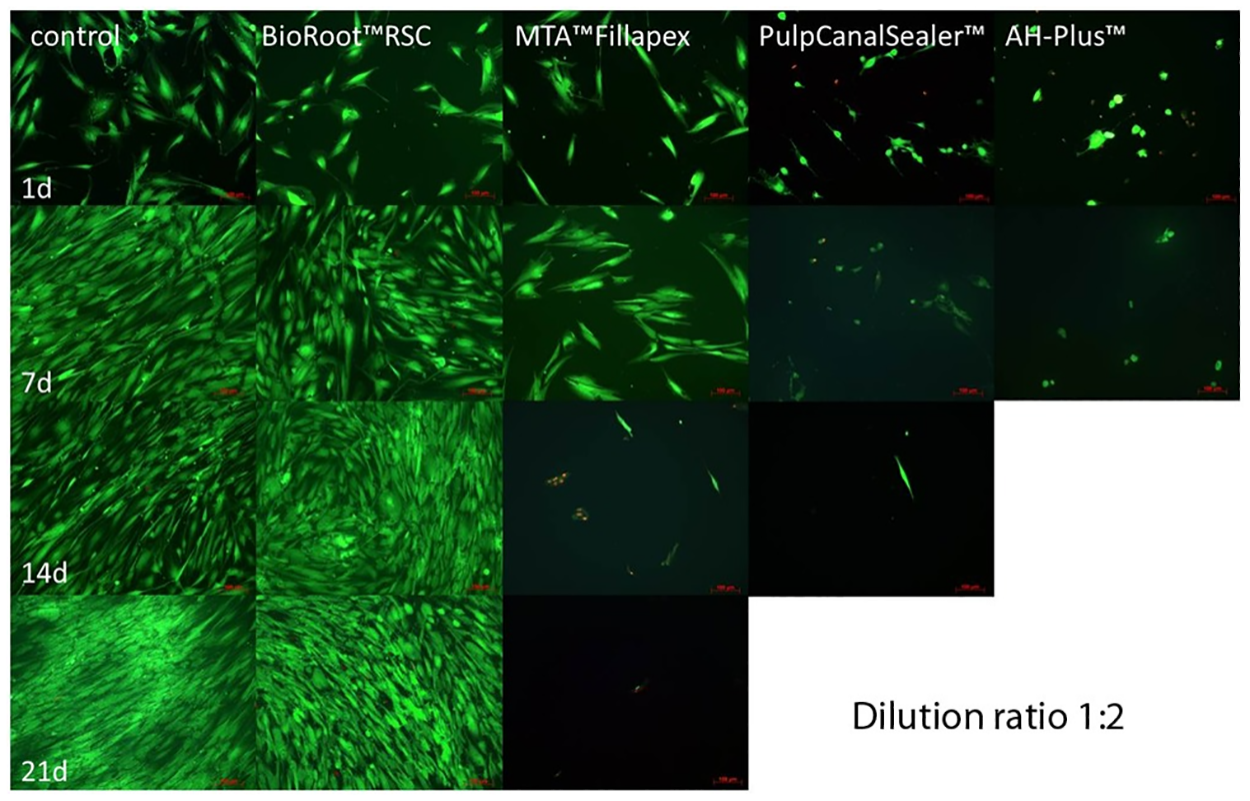
Living (FDA, green) / Dead (PI, red) staining (x100) of human osteoblasts after contact to different endodontic sealers (dilution 1:2) up to 21 d. (For AH Plus no living cells after 14 d and no picture for Pulp Canal Sealer after 21 d.)

**Fig 6:**
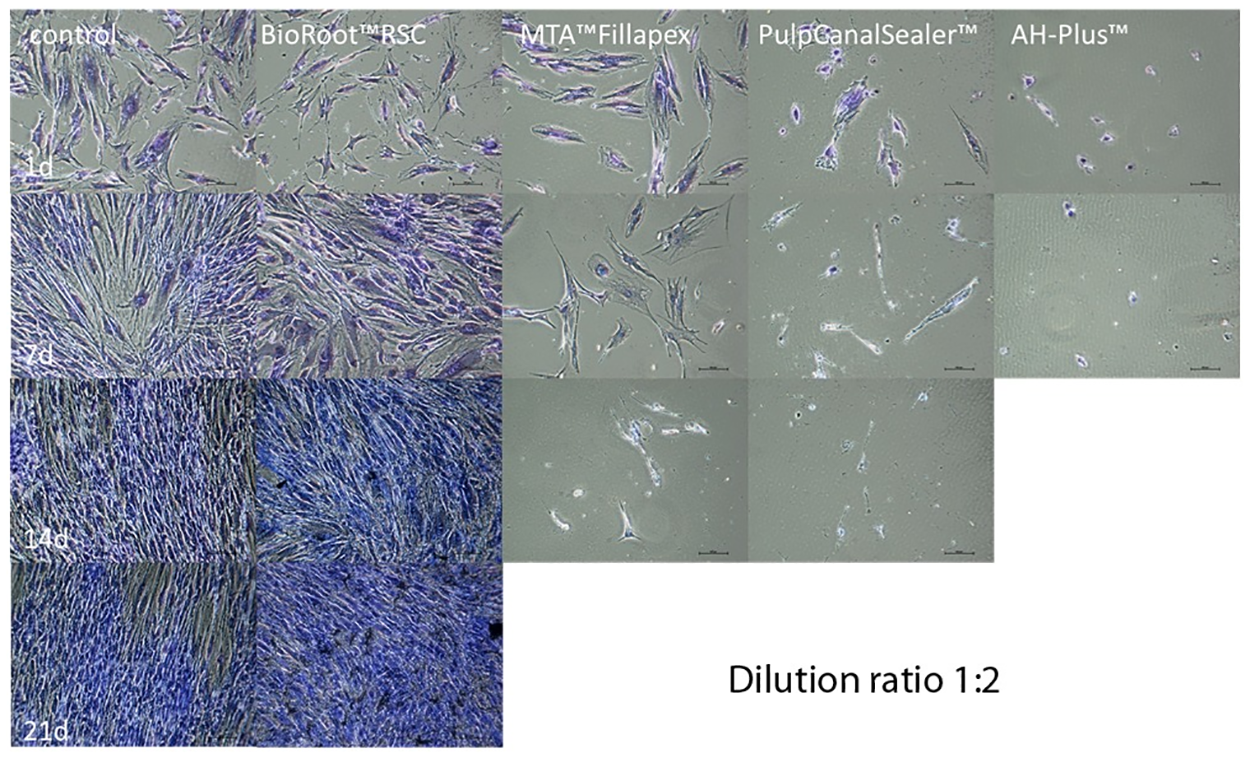
Richardson staining (x100) of human osteoblasts after contact to different endodontic sealers (dilution 1:2) up to 21 d. (For AH Plus no living cells after 7 days and no pictures for MTA Fillapex and Pulp Canal Sealer after 21 d.)

In the AH Plus (n = 24 cell tests) and the Pulp Canal Sealer group (n = 24 cell tests) all osteoblasts died during the first days after adding the sealer extract in a dilution of 1:2 (Figs 5 and 6). In the living/dead (Fig 5) and the Richardson staining (Fig 6) no or only a few cells were visible after 14 and 21 days, respectively. Furthermore, there was a high release of LDH with a factor of 2.99 for AH Plus and 2.85 for Pulp Canal Sealer during the first day (Fig 3).

During the first days in the MTA Fillapex group (n = 24 cell tests), the osteoblasts survived an extract diluted 1:2 (Fig 1). In the MTT assay, a conversion was observed until day 7 (Fig 2) and a LDH release until day 14 (Fig 3). In the living/dead (Fig 5) and the Richardson staining (Fig 6) there were nearly no living osteoblasts after 14 and 21 days, respectively.

In the BioRoot RCS group (n = 24 cell tests), all osteoblasts survived the contact with a 1:2 diluted extract and in contrast to all other sealers, a cell proliferation was observed (Fig 1). In the MTT assay of BioRoot RCS, the conversion was significantly higher compared to the control group (*p* < 0.05) except day 1 (Fig 2) but the number of living cells was lower (*p* < 0.05; Fig 1). A release of LDH was observable until day 21 (Fig 2). In the living/dead (Fig 5) and the Richardson staining (Fig 6) there were no differences compared to the control group.

**Fig 7:**
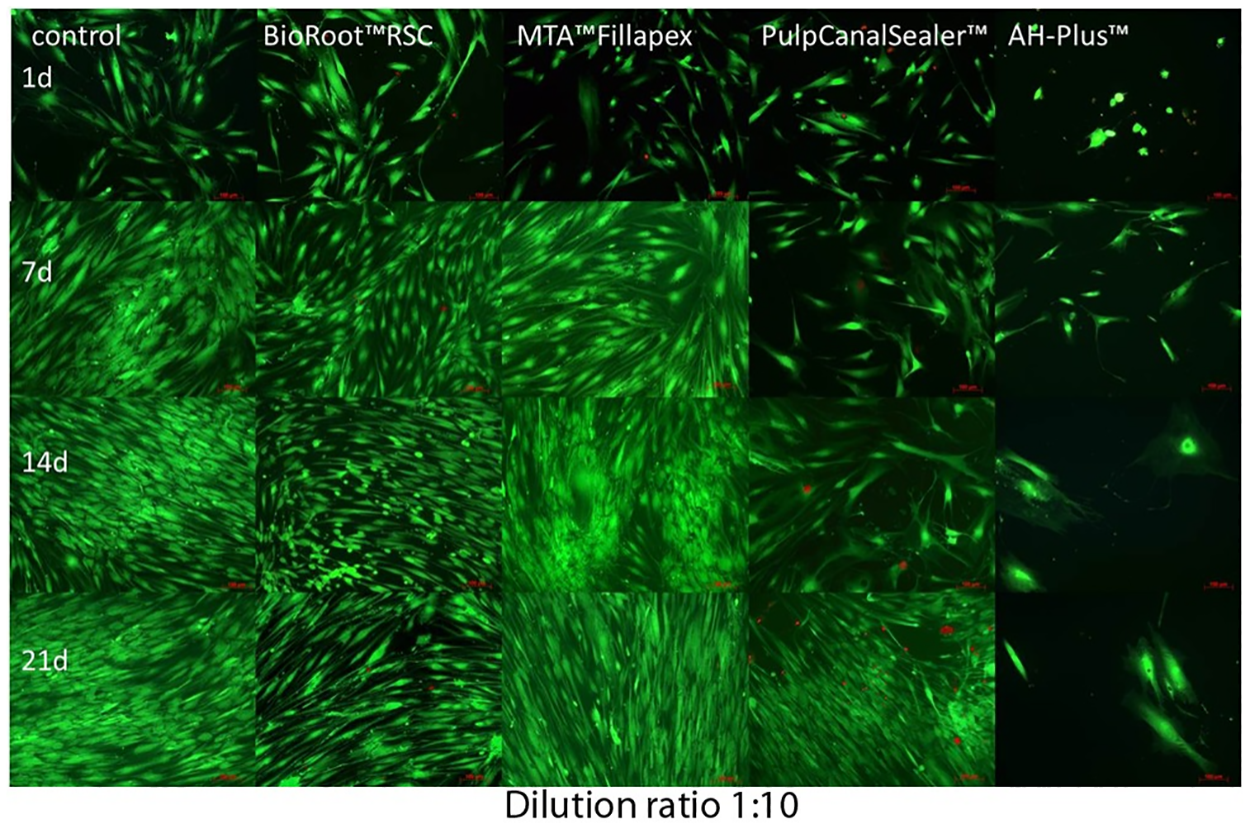
Living (FDA, green) / Dead (PI, red) staining (x100) of human osteoblasts after contact to different endodontic sealers (dilution 1:10) up to 21 d.

**Fig 8:**
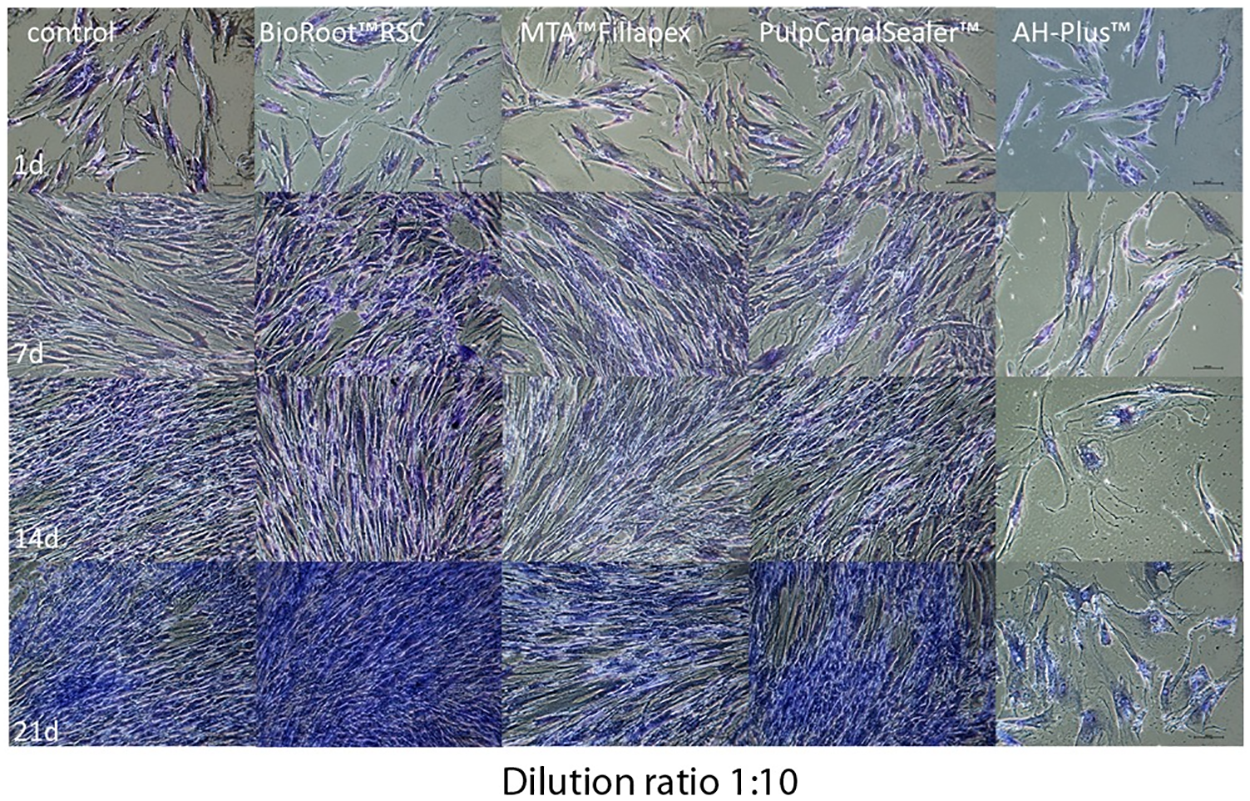
Richardson staining (x100) of human osteoblasts after contact to different endodontic sealers (dilution 1:10) up to 21 d.

In contrast to all other sealers, the osteoblasts did not survive the contact to a 1:10 diluted AH Plus extract (n = 24 cell tests) (Figs 7 and 8). In the living/dead (Fig 7) and Richardson staining (Fig 8) cells showed nearly the same low cell density. In contact to AH Plus the morphology of the osteoblast cells was altered; they become bigger with longer incubation period (Fig 8, days 14 and 21).

In a 1:10 dilution for all sealer (n = 96 cell tests) a release of LDH was observable and the release increased over time (Fig 4).

## Discussion

### Discussion of methods

Root canal sealers should be biocompatible as they can get into direct contact with periapical tissue through the apical foramen and accessory communications [21]. Thus, endodontic sealers should be selected not only on the basis of various physicochemical, but also on biological parameters, such as local biocompatibility. Only sealers with a satisfactory biocompatibility should be used for root canal obturation [5].

*Ex vivo* cell testing offers some information regarding the biocompatibility of new endodontic sealers in comparison with currently used ones. Hence, in the present study the reactions of human osteoblasts to four different sealers were evaluated in a freshly mixed and in a set state. A critical review of the literature reveals that the present study does not represent a novel approach. However, previous results—although interesting—are incomplete and insufficient to support their conclusions, e.g. all test models working only with set sealer samples have some limitations, because *in vivo* the surrounding tissue will be exposed to unset material.

Employing human primary cells of a relevant type in studies assessing endodontic materials has been pointed out previously [22] because primary cells derived from human target tissues of endodontic sealers, like human osteoblast cells from the alveolar bone or periodontal ligaments cells, are more relevant for biocompatibility studies than other cell lines [5]. For this, human osteoblast cells have been chosen for cytocompatibility testing here because these cells may get in direct contact with the sealer during root canal obturation. The specific setup for this *ex vivo* cell test was verified in a previous study [23].

So far the new calcium silicate based sealer BioRoot RCS was only tested on periodontal ligament [11–13] or mouse pulp-derived stem [14] cell lines. To the best of our knowledge this is the first study evaluating the reaction of human osteoblast cells to BioRoot RCS. Concerning MTA Fillapex only one study is currently available in the recent literature using human osteoblasts for cell testing [24].

The cytotoxicity and cell viability testing was performed only on chosen sealer’s extraction. It may be speculated that a direct method with direct contact of the cells to the materials may show different, more convincing results. But the aim of the present study was to simulate a more clinical scenario. It is well known, that in most clinical cases sealers have not a direct contact to the surrounding tissue. Rather, tissue fluid penetrates the root canal system and degrades the sealers. Then, leached substances may migrate to the periodontal tissues and alveolar bone, generate local periapical inflammatory reactions and adverse effects [3, 5, 6]–not the sealer itself. The direct contact will be investigated in a later study.

In the dilution 1:2 a high release of LDH (above 1) indicates that the cells had stress. In contact to AH Plus and Pulp Canal Sealer a high LDH value was only observed after 24 h, thereafter no longer. It can be concluded that these sealers were cytotoxic for the osteoblasts and after 7 d all cells were dead and thus LDH was no longer produced. Thus, the LDH release should only be interpreted in connection with other cell tests like living/dead or Richardson staining.

In the dilution 1:10, LDH was detected in an increasing rate. The sealer still caused stress in the cells but was not lethal. Clinically, inflammatory reactions may be caused by the release of cytokines.

### Pulp canal sealer

The cytotoxic and tissue-irritating potencies of zinc oxide-eugenol sealers were confirmed by the results of current publications on human periodontal ligament cells [11, 14], and on human osteoblasts [24], which are also in accordance with the present results. Zinc oxide eugenol type sealers are irritating mainly because of the eugenol [1]. Eugenol and other ingredients may modulate the immune response and contribute to periapical inflammation and pain [5].

### AH Plus

The results of the comparatively new sealers MTA Fillapex and BioRoot RCS were compared with AH Plus, an epoxy resin-based sealer, because AH Plus is one of the most widely evaluated sealer. Hitherto, it is known that AH Plus has excellent physical and sealing properties [15–17].

However, in the present study fresh specimen extracts of AH Plus exerted a marked cytotoxic effect. It was striking that in contact with eluates of AH Plus, osteoblasts became bigger (Figs 6 and 8). This can be interpreted as a pathological hydropic cell swelling and is a sign of degeneration [25].

AH Plus contains epoxy resin, which displays cytotoxic profile, especially at slightly diluted concentrations [26]. The epoxy resin present in AH Plus is mutagen and may cause breaks in the chain of cellular DNA [27]. According to the manufacturer, AH Plus is a formaldehyde free material. Nevertheless, a minute amount of formaldehyde release (3.9 ppm) was observed in a previous study [28]. This release of formaldehyde in combination with the release of amine and epoxy resin components may explain the cytotoxicity of freshly mixed AH Plus sealer [29].

Here, freshly mixed AH Plus was cytotoxic in a concentration-depending manner. This is in agreement with previous studies that have documented the cytotoxic effect of AH Plus immediately after mixing [29, 30]. Fresh AH Plus was strongly cytotoxic at a high extract concentration (1:2). After setting, AH Plus was no longer cytotoxic [29–31]. In contrast, other studies described AH Plus as moderately cytotoxic in fresh conditions, mildly cytotoxic after one week, and nontoxic after two weeks [32]. Compared to other resin-containing sealers, AH Plus showed the least cytotoxic effects, but lead to a reduction of cell viability of 26% [33]. AH Plus was 10 times more cytotoxic compared to BioRoot RCS [12]. On the other hand in an animal study with an induced apical periodontitis, the periapical tissues adjacent to root canals filled with epoxy-resin showed less inflammation compared to other sealers tested (zinc oxide eugenol and silicone) [34].

### MTA Fillapex

In contrast to AH Plus, MTA Fillapex remained severely cytotoxic over the entire experimental period, which is in accordance with other studies [31, 32]. Hence, MTA Fillapex may be described as more cytotoxic than AH Plus [31, 32]. Comparable to a zinc oxide eugenol sealer, MTA Fillapex exerted a negative impact on the viability of human dental pulp cells [35] as well as on human osteoblasts [24], and exhibited cytotoxic effects on osteogenic and angiogenic cells [8]. In an initial period MTA Fillapex was more irritating to bone tissue than AH Plus and did not improve bone tissue repair. Thus, MTA Fillapex is not bioactive [36]. Beside its severely cytotoxic effects MTA Fillapex remarkably decreased macrophages viability [6]. The severe toxicity of MTA Fillapex may be attributed to the presence of resinous components, mainly salicylate resin [6, 30–32, 36], which may induce apoptosis [37]. In the present study, the cytotoxicity of MTA Fillapex was related to the concentration of the eluates, which is in agreement with other authors [30].

### BioRoot RCS

In the BioRoot RCS group after 21 d significantly more cells were observed in comparison to the control group. According to MTT, BioRoot had a higher metabolic rate, i.e. more NAD(P), at low cell counts. Furthermore, BioRoot RCS showed a higher release of LDH than the controls over the entire time.

Hence, it may be concluded that BioRoot RCS had an influence on the cell metabolism and is only slightly cytotoxic. In agreement with recent studies [11–14] BioRoot RCS showed excellent biocompatibility at all extract concentrations as both fresh and set material. In direct contact with cells, BioRoot RCS was not cytotoxic and did not affect cell vitality and morphology. Cell growth was not adversely affected [11–14]. Concerning biocompatibility, in *ex vivo* cell tests BioRoot RCS showed better results than other sealers based on epoxy resin or methacrylate [12] or zinc oxide-eugenol based sealers [11, 14] and also better than other sealer based on calcium silicate [12, 13]. BioRoot RCS was the least cytotoxic sealer compared to other sealers with 98.54% cell survival, even when cells were treated with undiluted eluates [12]. In presence of set BioRoot RCS, human periodontal ligament cells showed a high degree of proliferation, cell spreading and cell attachment [13].

In contrast to Pulp Canal Sealer, BioRoot RCS did not compromise the osteo-odontogenic differentiation potential of pulpal A4 mouse pulpal stem cells, thus BioRoot RCS did not alter the viability and morphology of these cells. The intrinsic ability of A4 cells to express type 1 collagen, DMP1 or BSP was preserved [14].

In direct contact with human periodontal ligament cells BioRoot RCS showed bioactive effects and induced the secretion of angiogenic and osteogenic growths factors such as VEGF, FGF-2 and BMP-2 from the surrounding tissue, which influence the formation of blood vessels and bone [11].

During the first day in the BioRoot RCS group with a concentration of 4:1 an increase of the pH value to 11 could be observed. This high pH value may influence the material’s cytotoxicity and need to be investigated in another study.

### Cytotoxicity

The hypothesis had to be rejected. The results of the current study showed that the different sealers exhibited different levels of cytotoxicity. It must be remembered, however, that molecular leaching and therefore cytotoxicity might decrease over time. This depends on the solubility of the sealers. For instance, AH Plus is significantly less soluble than the here tested sealers MTA Fillapex and BioRoot RCS [18]. AH Plus is more or less insoluble after setting [16, 17], which explains that there was a marked difference in cytotoxicity between the freshly mixed and set specimens. Whereas unset samples of AH Plus showed a marked cytotoxicity, cytotoxicity was no longer observed in set AH Plus. MTA Fillapex is more soluble after setting than AH Plus [18], and this may explain the cytotoxicity of set and unset MTA Fillapex samples. Because of the solubility of BioRoot RCS, it may be speculated that BioRoot RCS is not only non-cytotoxic (biocompatibility) but may release some components to the surrounding tissue that might have a beneficial effect on tissue healing (bioactivity). Sealers with good biocompatibility are beneficial to aid or stimulate the repair of injured tissues.

It goes without saying, that the biocompatibility of a root canal sealer is only one of many factors that contribute to success of a root canal treatment. Overall, however, sealers based on calcium silicate can be regarded as an interesting alternative to conventional root canal filling materials.

## Conclusions

Within the limitations of this study, it can be concluded that with regard to “biocompatbilty” BioRoot RCS may be recommended for root canal obturation. Besides biocompatibility, BioRoot RCS is bioactive and had a positive influence on the cell metabolism. In contrast, contact of Pulp Canal Sealer and MTA Fillapex or freshly mixed AH Plus to osteoblasts should be avoided. Further investigations are necessary to prove the result of the present study.
